# Accuracy, interpretability and usability study of a wireless self-guided fetal heartbeat monitor compared to cardiotocography

**DOI:** 10.1038/s41746-022-00714-6

**Published:** 2022-11-03

**Authors:** Paul Porter, Huaqiong Zhou, Brooke Schneider, Jennifer Choveaux, Natasha Bear, Phillip Della, Kym Jones

**Affiliations:** 1 Department of Paediatrics, Joondalup Health Campus, Perth, WA Australia; 2 Faculty of Health Science, Curtin University, Perth, WA Australia; 3 Joondalup Health Campus, Partnerships for Health Innovation (PHI) Research Group, Perth, WA Australia; 4 Curtin University, Curtin School of Nursing, Perth, WA Australia; 5 Perth Children’s Hospital, Perth, WA Australia; 6 Institute for Health Research, Notre Dame University, Fremantle, WA Australia; 7 Department of Gynaecology and Obstetrics, Joondalup Health Campus, Perth, WA Australia

**Keywords:** Medical research, Diagnostic markers

## Abstract

Fetal Cardiography is usually performed using in-hospital Cardiotocographic (CTG) devices to assess fetal wellbeing. New technologies may permit home-based, self-administered examinations. We compared the accuracy, clinical interpretability, and user experience of a patient-administered, wireless, fetal heartbeat monitor (HBM) designed for home use, to CTG. Initially, participants had paired HBM and CTG examinations performed in the clinic. Women then used the HBM unsupervised and rated the experience. Sixty-three women had paired clinic-based HBM and CTG recordings, providing 6982 fetal heart rate measures for point-to-point comparison from 126 min of continuous recording. The accuracy of the HBM was excellent, with limits of agreement (95%) for mean fetal heart rate (FHR) between 0.72 and −1.78 beats per minute. The FHR was detected on all occasions and confirmed to be different from the maternal heart rate. Both methods were equally interpretable by Obstetricians, and had similar signal loss ratios. Thirty-four (100%) women successfully detected the FHR and obtained clinically useful cardiographic data using the device at home unsupervised. They achieved the required length of recording required for non-stress test analysis. The monitor ranked in the 96–100^th^ percentile for usability and learnability. The HBM is as accurate as gold-standard CTG, and provides equivalent clinical information enabling use in non-stress test analyses conducted outside of hospitals. It is usable by expectant mothers with minimal training.

## Introduction

The fetal heart rate (FHR) and heart rate variability are used as indicators of fetal wellbeing in utero. During routine low-risk antenatal consultations, the fetal heart rate is measured briefly by a handheld doppler device, a DeLee-Hillis stethoscope, or a Pinard horn, depending on available skills and resources. This process is known as intermittent auscultation (IA)^1^.

In high-risk pregnancies and emergencies, a Non-Stress Test examination (NST) is performed using cardiotocography (CTG) to collect comprehensive FHR data (fetal cardiography) for >10 minutes and to assess uterine contractions (tocography)^2^. FHR and movement data are combined to estimate fetal hypoxia at the time of the test, reported as reactive or non-reactive (definitions and assessment criteria: Box 1), which is used to judge fetal health and guide clinical interventions.

We have previously described using a FHR monitor for intermittent auscultation by clinicians and when self-administered by women^3^. HeraBEAT (HeraMED, Netanya, ISRAEL) is a medical-grade, low-cost, wireless, self-guided fetal and maternal heartbeat monitor (HBM) designed for self-administration from 12 weeks of gestation.

Our previous research showed that when this HBM was used in low-risk pregnancies, the results were accurate and as clinically useful as those obtained by handheld fetal Dopplers. In addition, the device was easy to use^3^. However, this research used FHR traces of less than 5 min duration and did not investigate the suitability of using the HBM for the longer recordings required for NST examinations (10–20 min).

To introduce home-based fetal cardiography monitoring into the standard care model, we need to ensure the device is equivalent to the clinic standard CTG device in terms of accuracy and clinical interpretability and to show equivalence when used by women at home unsupervised.

Since the COVID-19 pandemic, the introduction of telehealth consultations into maternity care has become standard^4,5^. The addition of telehealth allows women to access antenatal care remotely without causing detrimental maternal and fetal outcomes^5,6^. However, certain crucial aspects of antenatal care have so far been hard to deliver in a telemedicine antenatal care model^4^.

Tocography is easily performed directly by women through simple available technology. However, accurate and reliable measurement of FHR outside clinical environments has proven more difficult. Patient-delivered FHR monitors are available, but there have been issues with usability, accuracy and reliability, signal noise, differentiation of fetal from maternal heart rate (MHR), inadequate recording duration, and cost^7^. Handheld Doppler devices used in clinics require training to operate, cannot differentiate between FHR and MHR, and cannot store or transmit data. There have been attempts to use mobile CTG machines; however, these machines are costly and not easily transportable. For home monitoring to be practical and clinically useful, FHR monitors need to be as accurate as CTG, provide data that clinicians can interpret, be self-administered, and allow secure and reliable data transmission.

This study has two aims. Firstly, we compare the point by point (FHR) accuracy and the clinical interpretability of a 20-minute fetal cardiography examination obtained using a wireless mobile self-guided fetal heartbeat monitor to one obtained from a traditional CTG stationary device when used in the clinic environment. Secondly, we evaluate the HBM in terms of clinical utility, usability, and learnability when used by women, unsupervised, and at home.

Box 1 Definitions of the fetal heart rate characteristics required to assess fetal cardiography and non-stress test examinations^21,22^ Fetal heart rate characteristics used to assess fetal cardiography:

Baseline (bpm): Visual determination of the mean level of the ‘resting’ heart rate (not sleeping heart rate) and is assessed in the absence of fetal accelerations, decelerations, and contractions. The baseline rate is the mean bpm (rounded to 0 or 5) over a 10-minute interval, excluding periodic changes, periods of marked variability, and segments that differ by more than 25 bpm. The baseline must be identifiable for two minutes during the interval (but not necessarily a contiguous two minutes); otherwise, it is considered indeterminate. The normal baseline rate ranges from 110–160bpm.

Accelerations: An abrupt increase in the FHR. Before 32 weeks of gestation, accelerations should last ≥10 sec and peak ≥10 bpm above baseline. As of 32 weeks gestation, accelerations should last ≥15 sec and peak ≥15 bpm above baseline. A prolonged acceleration is ≥2 minutes but less than 10 minutes. An acceleration of 10 minutes or more is considered a change in baseline.

Decelerations: Transient episodes of FHR below the baseline of more than 15 bpm lasting at least 15 seconds. They are classified as early, late or variable types.

Variability: Minor 3–5 cycle per minute fluctuations around the baseline FHR. It is visually assessed by estimating the difference in beats per minutes between the highest peak and the lowest trough of fluctuations at the baseline and is assessed over 1 minute segments.

Variability grade: Absent: amplitude undetectable, minimal: amplitude 0 to 5 bpm, moderate: amplitude 6 to 25 bpm, marked: amplitude over 25 bpm.

Non-Stress Test Evaluation guidelines:

The American College of Obstetrics and Gynecology has given guidance in the evaluation of fetal heart rate characteristics during pregnancy. In the nonstress test, the heart rate of a fetus that is not acidotic or neurologically depressed will temporarily accelerate with fetal movement. Heart rate reactivity is believed to be a good indicator of normal fetal autonomic function. Loss of reactivity is commonly associated with a fetal sleep cycle but may result from any cause of central nervous system depression, including fetal acidosis.

Nonstress tests are classified as reactive or nonreactive. Various definitions of reactivity have been used. Commonly, the nonstress test is considered reactive, or normal, if there are two or more fetal heart rate accelerations within a 20-minute period. The FHR of a neurologically healthy preterm fetus is frequently nonreactive. The presence of decelerations and decreased variability are signs of potential fetal compromise.

## Results

### Study enrolment

**Fig. 1:**
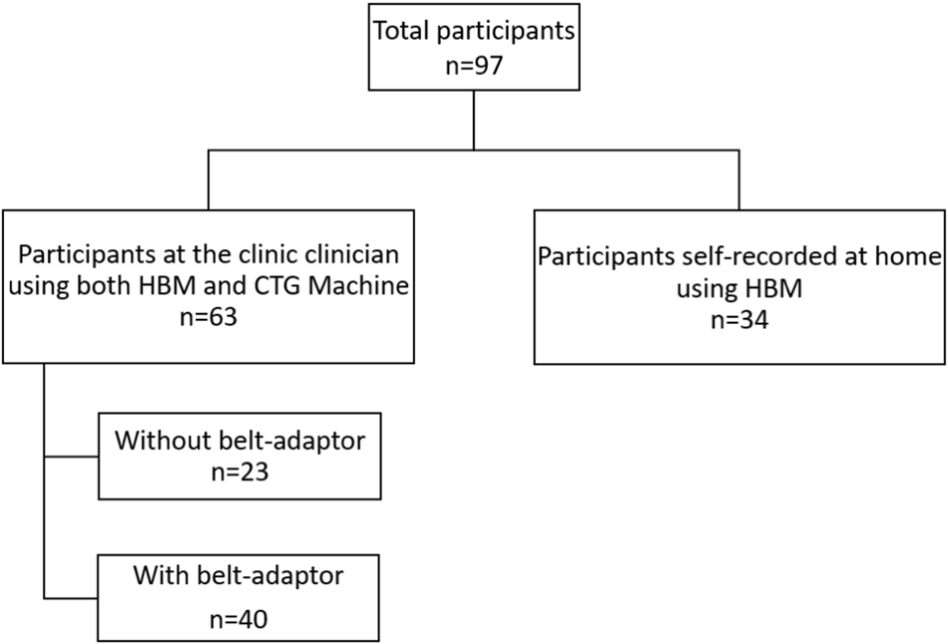
Participants flow through the study. Heartbeat monitor (HBM), Cadiotocograph (CTG).

Between November 2020 and August 2021, we enrolled 97 women in the study from the antenatal clinic of a large metropolitan hospital in Western Australia (Fig. 1). The study consisted of two phases. Phase 1 (November 2020 to July 2021) was undertaken in the clinic and inpatient ward of the recruiting site, with clinicians conducting both the CTG and HBM examinations. Phase 2 (March to August 2021) was undertaken at home, where study participants used the HBM as a self-monitoring device.

Phase 1: Clinic-based monitoring––comparison of accuracy and clinical interpretability

In this phase, we compared the beat-to-beat accuracy of the HBM and CTG using paired measurements. A total of 63 women were recruited, generating 6,982 pairs of data points for analysis. Twenty-three participants did not use a belt to stabilize the HBM (2,562 data points), while 40 used a belt (4,420 data points). The FHR was detected, the baseline confirmed to be in the normal range, and noted to be different from the MHR on 100% of occasions using the HBM. No adverse events were reported.

The characteristics of the study participants are presented in Table 1. The age range was 20 to 39 years with a mean of 30.5 ± 4.6, of which 43 were aged 26–30. Gestational age at enrolment was in the third trimester and ranged from 29.4 to 40.9 weeks, with BMIs ranging from 22.8 to 41.8 kg/m^2^ (mean 31.0 ± 4.2). Twenty-seven participants (42.9%) had BMIs from 23.5 to 29.9. Pre-pregnancy BMIs ranged from 17.4 to 35.2 with a mean of 25.9 ± 4.0, and more than half (*n* = 34) had BMIs between 23.5 and 29.9, and 15.9% were in the obese category (BMI > 35). Thirty-five participants (55.6%) had more than two pregnancies, and 30 (47.6%) had one previous live birth. Almost 50% of participants had an anterior placenta (*n* = 31), with 46% having a posterior placenta (*n* = 29).

**Table 1:** Demographic and clinical characteristics of Phase 1 (clinic monitoring) participants.

Variables	Lowest	Highest	Mean	SD
Age (year)				
Total cohort	20	39	30.5	4.6
Counts/Percentage				
≤25	7	11.2%		
26–30	27	42.8%		
31–35	19	30%		
≥36	10	15.9%		
Gestational age (week)				
Total cohort	29.4	40.9	37.7	2.8
BMI at Enrolment (kg/m^2^)				
Total cohort	22.8	41.8	31.0	4.2
Counts/Percentage				
Lower than 23.5 (Counts/Percentage)	2	3.2%		
23.5–29.9	27	42.9%		
35–34.9	24	38.1%		
45 or higher	10	15.9%		
BMI Pre-pregnancy (kg/m^2^)				
Total cohort	17.4	35.2	25.9	4.0
Counts/Percentage				
Lower than 23.5	19	30.2%		
23.5–29.9	34	54%		
30–34.9	8	12.7%		
35–44.9	2	3.2%		
Gravidity				
Counts/Percentage				
1	28	44.4%		
2	15	23.8%		
≥3	20	31.8%		
Parity				
Counts/Percentage				
0	33	52.4%		
1	20	31.7%		
2	6	9.5%		
≥3	4	4.8%		
Placenta Location				
Counts/Percentage				
Anterior	31	49.2		
Posterior	29	46		
Other	3	4.8		

The cohort’s average FHR was 141.5 beats per minute (bpm), and the average MHR was 87.4 bpm. The fetal heart rate was detected quickly, with the time taken to first detection ranging from under 15 s to 1 min (*n* = 62), with one outlier at 6 minutes (*n* = 1). The average duration of fetal cardiography recorded was more than the required time needed for NST examination analysis, 32.4 ± 12.1 minutes, and ranged from 16 to 78 minutes.

The longest continuous HBM collected cardiography segment (without an episode of transient signal loss) for all participants ranged from 2.4 to 48.8 minutes, with an average of 10.7 minutes. A positive influence was seen from wearing a stability belt with the longest continuous segment obtained for the “no belt” group ranging from 2.4 to 25.3 minutes (average 9.6 minutes) and for the “with belt” group ranging from 2.9 to 48.8 minutes (average 11.3 minutes).

Signal loss is an essential measure of the quality of fetal cardiography. Less than 20% signal loss is required for a trace to be acceptable. The mean signal loss was 15.0% for the HBM “no-belt” group compared to 3.0% for the CTG and 8.5% for the HBM “with belt” group compared to 3.4% for the CTG. The “with belt” group had significantly less signal loss than the “no-belt” group (*p* = 0.004).

There was no association between pregnancy variables, including BMI, gestation, placental position, or recording site, and the time taken to detect an FHR, trace duration, or signal loss ratio (supplementary table 4). In particular, even in women with potential anatomical barriers to ultrasound, such as having an anterior placenta (*n* = 31), or a BMI of 35 to 44.9 kg/m² (*n* = 10), there was no difference in FHR detection and trace duration.

To compare the accuracy of fetal cardiography achieved by the two methods, we defined ± 8 bpm as an acceptable difference based on published literature^8,9^. The accuracy of the HBM compared to the CTG device was excellent and well below the specified acceptable level. When we used all paired data points, we found the mean difference between 0.53 and 0.63 bpm. The 95% limits of agreement (LOA) mean difference, intraclass coefficients, and the number of individual pairs outside of the LOA are shown in Table 2.

**Table 2:** Fetal heart rate agreement between heartbeat monitor and cardiotocography for all paired time points, and for participants who used and did not use a stability belt. All measures in beats per minute.

Heartbeat monitor usage	Number of paired FHR measurements	Mean difference (SD)	95% limits of agreement	Number (%) of pairs outside 95% limits of agreement	Intraclass coefficient (95% CI)
All participants	6982	0.53 (2.45)	−4.27 to 5.32	259/6982 (3.7%)	0.970 (0.968–0.971)
Participants who used a belt	4420	0.63 (2.48)	−3.90 to 5.17	182/4420 (4.12%)	0.968 (0.966–0.970)
Participants who did not use a belt	2502	0.31 ± 1.8	−4.89 to 5.13	92/2562 (3.59%)	0.970 (0.967–0.972)

Three further Bland Altman analyses were conducted using the average beat-to-beat difference from each participant. Firstly, all participants were analyzed (*n* = 63) and then subdivided further into those who used a belt (*n* = 23) and those who did not (*n* = 40).

**Fig. 2:**
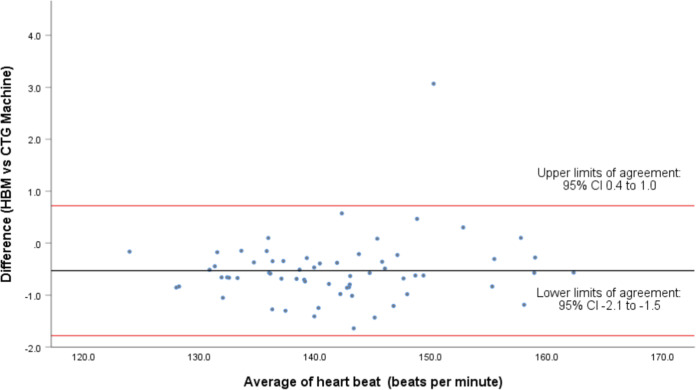
Bland-Altman plot showing difference in heart rate (beats per minute) between 63 pairs of fetal cardiographs recorded by the fetal heartbeat monitor and cardiotocography. The difference in mean fetal heart rates for each participant was calculated using all data (*n* = 6982).

A comparison of the means of all measured time points for the total group (*n* = 63) showed 95% LOA of 0.72 (CI 0.4 to 1.0) and −1.78 (CI −2.1 to −1.5), with a mean difference of −0.53 ± 0.64 bpm (Fig. 2). The intraclass coefficient was excellent at 0.998 (95% CI 0.987 to 0.999). The linear regression/coefficient results indicated no presence of proportional bias (*p* = 0.278).

**Fig. 3:**
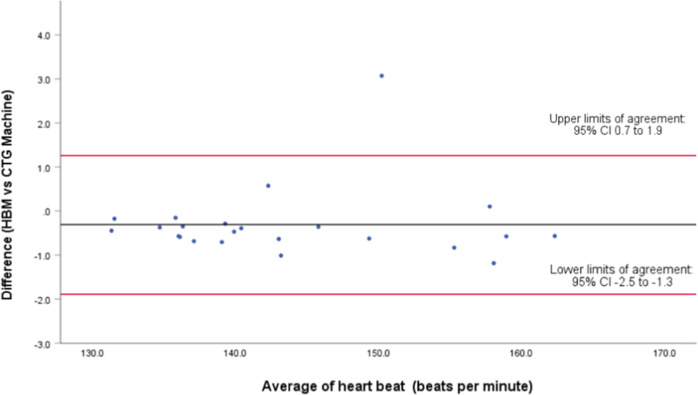
Bland-Altman plot. Difference in heart rate (beats per minute) between 23 pairs of cardiographs recorded by the fetal heartbeat monitor without-belt stabilisation and cardiotocography.

For participants with “no-belt” (*n* = 23), the difference between the means of measured time points showed 95% LOA of 1.26 (CI 0.7 to 1.9) and −1.89 (CI −2.5 to −1.3), with a mean difference of −0.31 ± 0.804 bpm (Fig. 3). The intraclass coefficient was 0.998 (95% CI 0.995–0.998). The linear regression/coefficients results indicated no presence of proportional bias (*p* = 0.857).

**Fig. 4:**
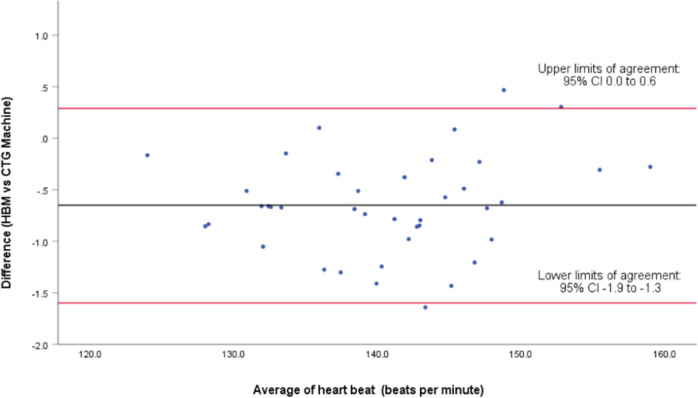
Bland-Altman plot. Difference in heart rate (beats per minute) between 40 pairs of cardiographs recorded by the fetal heartbeat monitor with-belt stabilisation, and cardiotocography.

For those “with belt” (*n* = 40), the difference between the means of measured time points showed 95% LOA of 0.29 (CI 0.0 to 0.6) and −1.6 (CI −1.9 to −1.3), with a mean difference of −0.65 ± 0.48 bpm (Fig. 4). The intraclass coefficient was 0.997 (95% CI 0.931–0.999). The linear regression/coefficients results indicated there is no presence of proportional bias (*p* = 0.285).

A specialist obstetrician assessed all recordings from the HBM and the CTG machine in random, de-identified order to determine the adequacy of the traces for clinical analysis. There was no attempt to grade the traces as clinically healthy (reactive or non-reactive) but only to decide the suitability for analysis. After this procedure, we superimposed HBM and CTG traces for visual comparison (Fig. 7 shows a typical comparison).

The baseline FHR was determined in all HBM and CTG recordings. No significant difference was found between the devices, with a mean difference of 1.41 ± 2.79 (*p* = 0.95).

Twenty of all 63 pairs (31.7%) had differing baseline measurements with a non-significant mean difference of 1.26 ± 2.14 (*p* = 0.79, 95% CI 0.70 to 1.82). Six of the 23 “no belt” pairs (26.1%) also had a small but non-significant difference in the FHR baseline. Fourteen of the 40 participants in the “with-belt” group (35%) had a similar non-significant difference of 1.17 ± 1.67 (*p* = 0.63).

Fifty-five of the 63 (87.3%) paired recordings were deemed acceptable for measuring accelerations. Of the 8 HBM recordings considered less interpretable, loss of signal (LOS) during the period when accelerations were noted on the CTG was the cause. However, the assessment was equivalent between devices when the signal was maintained. Prolonging the HBM recording time, as is recommended in NST protocols, would have potentially converted these eight recordings into equivalence; however, this was not possible using the study methodology as the time for recording was determined by clinical staff based on the CTG review alone.

All sixty-three paired recordings (100%) could be used in assessing the presence of variability. In 60 (95.2%), the degree of variability was graded equally. For three (7.5%) participants who used a belt, the variability was graded as reduced on the HBM compared to the CTG trace. One participant displayed FHR decelerations on both the HBM and CTG recordings. Decelerations indicate fetal compromise and the need for urgent clinical care. This finding was unexpected given the clinical setting and resulted in an augmented delivery of the infant.

Phase 2: Home monitoring

Thirty-four women participated in this phase of the study. The ages ranged from 21 to 39 years, with a mean of 31.4 ± 4.9, of which 26% were between 31 and 35. The gestational age at enrolment ranged from 22.9 to 41, with a mean of 35.0 ± 4.3. Enrolment Body Mass Index (BMI, kg/m^2^) ranged between 22.6 to 42.3 kg/m² (mean 32.4 ± 4.6), with 9 (22.5%) BMIs between 23.5 and 29.9 kg/m². Pre-pregnancy BMIs ranged from 20.7 to 40.8 kg/m² (mean 27.0 ± 4.3), with over half (*n* = 21) having BMIs between 23.5 and 29.9, kg/m² and 29% (*n* = 10) being >35. Twenty-one (61.8%) women had had more than two pregnancies, and 28 (82.6%) had had one live birth. Almost 56% (*n* = 19) had anterior placentas and 35% (*n* = 12) had posterior placentas (35%, *n* = 12).

All traces recorded at home (34/34) could be assessed and provided clinically appropriate data. The FHR was detected and identified as separate from the MHR by every participant. All data was successfully transmitted electronically via the APP interface to the clinic staff for review. The average FHR of the group was 137.7 bpm (95% CI 135.0–140.4), while the MHR was 88.9 bpm (95% CI 85.9–91.9). Participants found the FHR quickly, with an average time to detection of 48.2 seconds ±2.11 minutes, ranging from less than 15 seconds to 12.5 minutes. The average total recording duration was more than that required for NST assessments, with an average of 28.5 minutes (range from 20 minutes to 56.9). The signal loss was under the required level, with a mean of 6.7 ± 5.9%.

We used the international medical standard System Usability Scale (SUS) to determine the usability and learnability of the HBM. Twenty-five of the 34 participants (73.5%) returned online SUS questionnaires. Participants found the device easy to use, with the mean total, usability, and learnability scores ranked in the 96th–100th percentile (Table 3). The Cronbach Alpha was 0.81, which indicates good internal reliability. An additional adjectival rating scale scored on a Likert scale of 1–7 gave a median score of 6.

**Table 3:** System Usability Scale Results of Women who used heartbeat monitor unsupervised at home (phase 2).

System Usability Scale Results for Women using heart beat monitor, unsupervised at home			
Grading Metric	Total Score	Usability	Learnability
Raw SUS score	84.4 (79.6–89.3)	83.9 (78.3–89.5)	86.5 (80.6–92.4)
Percentile ranking	96–100	96–100	96–100
Graded score (A+ to F)	A+	A+	A+
Adjectival rating scale (0–7)	6 (6–7) [4–7]		

SUS, System Usability Scale. Raw SUS scores are mean (95% CI), and adjectival rating scale are median (interquartile range) unless otherwise specified.

There was no association between pregnancy variables, including BMI, gestation, placental position, or recording site, and the time taken to detect an FHR trace duration or signal loss ratio (supplementary table 5). In particular, FHR detection and trace duration was not compromised for women with the potential anatomical barriers to ultrasounds of having an anterior placenta (*n* = 19) or a BMI over 45 kg/m^2^ (*n* = 10) at enrolment of study.

## Discussion

The results of this study show that the HBM is comparable to the current standard of care hospital-based CTG machines, and in addition, the device is easy to use for pregnant women outside of a clinical setting.

The HBM and CTG were equivalent in beat-to-beat accuracy and in producing data for assessment parameters essential to fetal cardiography grading during NSTs, including determining the baseline FHR, accelerations, decelerations, and variability. In addition to being easy to use at home without the need for clinical supervision, data delivery to the clinical team was secure and reliable.

The robust data obtained, along with the HBM’s portability and self-guiding facility, means that the device can be used for remote and home monitoring. This facility has not been possible before. This finding allows for more equitable, accessible, and convenient fetal monitoring compared to using CTG machines which require attendance at a clinic and a trained clinician operator and is costly. The HBM provides a new level of clinical rigour and safety for integrated antenatal care models involving virtual and face-to-face consultations. In addition to home monitoring, this approach may result in better engagement and empowerment of women in their pregnancies.

In contrast with our previous work, which assessed data over a series of 5 paired time points, this study analyzed the FHR data over a 20-minute session, consistent with the requirements for NST examinations. Using a more robust methodology and significantly more points of comparison (*n* = 6982), we found that the FHR was well within the predefined acceptability limit of ±8 bpm. This result confirms our previous findings, which were measured over shorter time frames. Given advances in fetal cardiography accuracy, such as shown with this HBM, we suggest that the predefined accuracy limits be reduced for future accuracy studies from the currently accepted level of ± 8bpm.

The HBM produced equivalent data to the CTG for three of the required heart rate parameters, with 100% agreement that Baseline is determinable, Deceleration is determinable, and Variability is determinable. The remaining two parameters, determinability of accelerations and variability, showed agreement in 87.3% and 95.2%, respectively. Each episode of disagreement was due to LOS. Due to the methodology used where the CTG monitor was placed in the prime recording position, this loss of contact from the HBM may have resulted from sub-optimal positioning; however, further work is needed to clarify this observation.

Signal loss ratio (SLR) is an important quality control measure in NST examinations. Current standards require the SLR to be less than 20%. The SLR was 15% when the HBM was used without a belt, and 8.5% when used with a belt are less than the predefined limit; however, is more than that obtained with the CTG device (3–3.4%). This effect was seen as a decrease in the detection of accelerations and in the grading of variability in a minority of cases. In clinical practice, the cardiography would be continued until the FHR characteristics are seen. A non-reactive trace at 20 minutes (less than two accelerations seen) is continued until the clinical picture is more apparent. Recordings are typically extended by another 20 minutes to separate the fetus in a period of prolonged quiet sleep from those who are hypoxemic or asphyxiated^10^. Our methodology did not allow HBM recordings to continue longer than the time required by clinical need which was determined by the clinical team’s assessment of the CTG trace. Longer recordings may have resolved the minor observed discrepancy in accelerations and grade of variability.

Various anatomical factors can limit ultrasound examinations. However, we found that neither BMI (including obese participants) nor the presence of an anterior placenta changed the accuracy, SLR, or interpretability of the HBM.

The use of a belt did not affect the device’s accuracy; however belts were associated with a decrease in SLR. The device is designed for self-administration, but there are practical hurdles to holding a device in the same position for 20 minutes. Therefore, while the beat-to-beat accuracy of the HBM can be relied upon with or without a belt, the belts are inexpensive and disposable and it is recommended that one be used to maximize clinical efficiency and reduce the time needed to record all clinically relevant data.

While phase 1 of the study showed that clinician use of the device is achievable, the intended clinical application is for women to use it at home without supervision Successful self-administration would support the use of the HBM in circumstances where expert care is not available. Phase 2 evaluated the usability and learnability of the device when used after a short 5-minute education session. As with our previous results, all participants were able to quickly detect the fetal heart, and then obtain recordings of adequate length and quality for subsequent NST analysis. Interestingly, the SLR was 6.7%, less than that observed in the clinician-applied HBM group of Phase 1 (8.5%, all participants in phase 2 used a belt). Participants ranked the HBM highly in terms of usability and learnability (96–100% percentile), confirming findings from our earlier study^3^.

This study has several limitations including a study population that was recruited from a single center, and excluded non-English readers, as well as those without smartphone access. However, we do not expect that language will not be an obstacle to broader use as positional guidance is provided verbally by the smartphone interface and is available in multiple languages. Although the number of participants was relatively small; we had a large number of paired FHR data points, which means the results should be more robust. This study’s accuracy and usability results are consistent with previously published work^3^. A further trial with a larger participant sample size in a formal clinic situation to determine scalability would be helpful. Although not an aim of this current study, consideration should be given to using the HBM in real-time to allow clinicians to continue recording long enough to formally characterize the traces as reactive or non-reactive NSTs.

It is important to note that the HBM is an FHR monitor only and does not monitor uterine contractions or fetal movement. This is relevant as both are monitored during NSTs. The lack of tocography limits the ability to determine the nature of FHR decelerations. However, in the absence of decelerations, the requirement for tocography is lessened if normal variability and acceleration characteristics are observed as they are highly correlated with adequate fetal oxygenation. Several low-cost methods exist for monitoring fetal movement that could be incorporated into the system, including the user tapping on the smartphone when movements are felt or using a disposable tocographic belt. CTGs are also used during labor, where a contraction monitor is desirable. It should be noted that we have not examined the HBM during labor, and it is not recommended to be used in this situation until formally assessed.

The use of remote monitoring and telehealth in maternity care has been limited by the difficulty in monitoring the developing fetus outside clinical environments^4^. While it is relatively easy to monitor maternal physical and mental health, fetal monitoring has been hampered by a lack of FHR monitors that women can safely use at home^2,7,9,11–13^. One significant concern has been the potential for confusion between the detection of MHR and FHR. A healthy FHR is significantly higher than MHR. When the FHR falls into the normal range for MHR, it indicates significant fetal hypoxia and requires emergency attention. Any home-based monitor must always be able to separate FHR from MHR. The HBM used in this study is designed to differentiate FHR from MHR, and this was shown for all 184 study participants. The device will not report a FHR unless both the MHR (via the optical sensor) and the FHR (via ultrasound) are detected at the same time.

Many obstetric services introduced Telehealth consultations into routine antenatal care during the COVID-19 pandemic for infection control reasons. Maternal COVID-19 infection, particularly in unimmunised women, is associated with poor maternal and neonatal outcomes. As a result, the Royal Australian and New Zealand College of Obstetrics and Gynaecology recommended reducing face-to-face visits, limiting consultations to less than 15 minutes, and substituting telehealth consultations (https://web.archive.org/web/20220114144756/https://ranzcog.edu.au/statements-guidelines/covid-19-statement/information-for-pregnant-women). Resistance to these models of care may stem from concerns over the adequacy of fetal monitoring and perceptions that clinician engagement is tied to personal interactions^14^. The robust surveillance of maternal and fetal biomarkers is essential for telehealth programs to be effective^14^. The accuracy, clinical interpretability and usability of the HBM, as well as its ability to store and transmit data may offer confidence to clinicians and women when transitioning towards a telehealth service model incorporating home-monitoring^13^.

The HBM device can be used in remote medicine. During the Russian-Ukraine war, the device was used by women who were unable to attend clinics due to safety reasons. The recordings were analyzed by obstetricians in Israel. This model deserves further investigation and development for scenarios where clinical care or CTG is not available. In addition, a role during labor should be investigated. Globally, up to 2 million fetuses die during labor each year^15^. The International Federation of Gynecology and Obstetrics recommends intermittent auscultation when there is no access to cardiotocography machines^16^. In resource-limited settings, the HBM monitor would allow inexperienced operators to accurately record, store, and transmit much longer and clinically useful FHR data. The HBM connects to a web-based platform allowing clinicians to review the FHR trace in real time during remote recording. Further research is required to determine whether the monitor remains accurate during contractions.

## Methods

### HBM Operating parameters

**Fig. 5:**
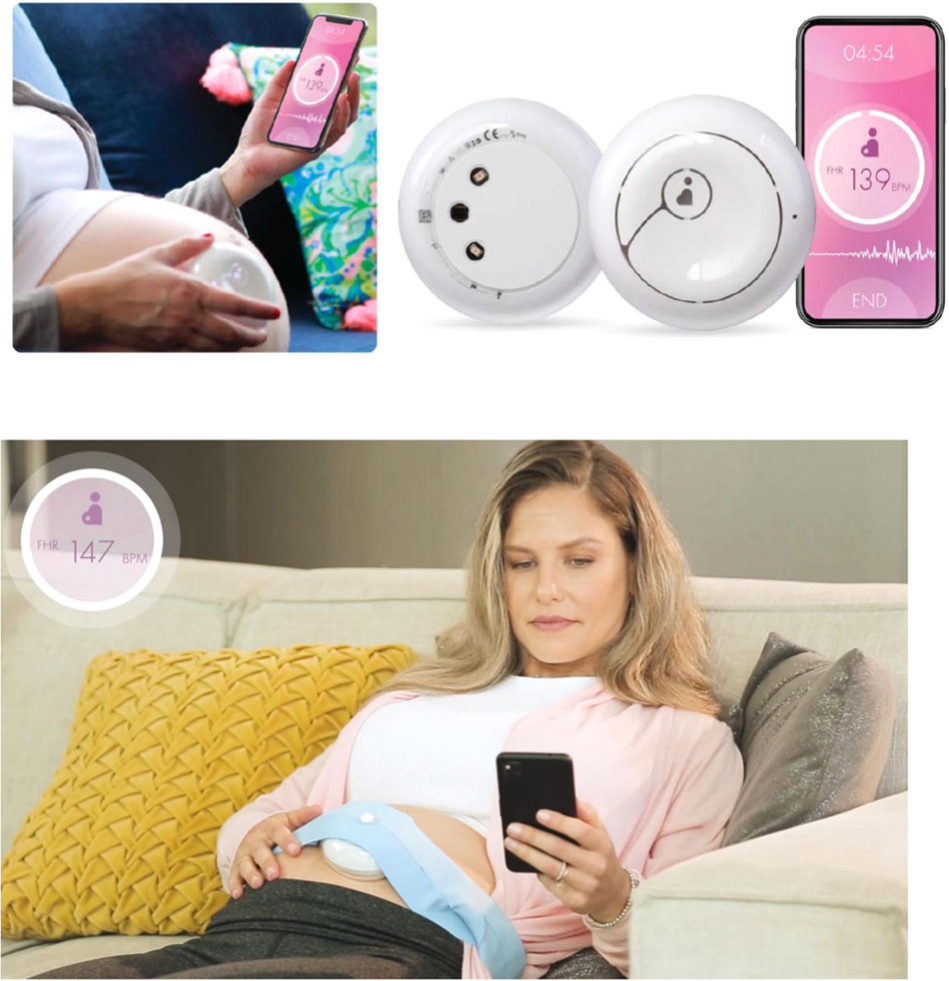
Fetal heart rate monitoring system. Device and integrated smartphone interface. Hand held and with disposable stability belt Image (courtesy of HeraMED Ltd. Used with permission).

The HBM weighs 130 g, is 9 cm in diameter, and is designed for use by non-clinical operators (Fig. 5). The HBM employs ultra-wide beam Doppler technology to measure the FHR and integrates a dedicated optical sensor to directly monitor the MHR from the abdomen, eliminating FHR–MHR cross talk.

The device is activated, coated with ultrasound gel, and placed below the umbilicus, directed by a smartphone interface, to a position dependent on pregnancy gestation. The device continues to self-direct positioning using audio instructions until two distinct heart rates (FHR and MHR) are detected. Then, clinicians can use a manual method to place the device directly in the appropriate position on the abdomen without voice guidance.

**Fig. 6:**
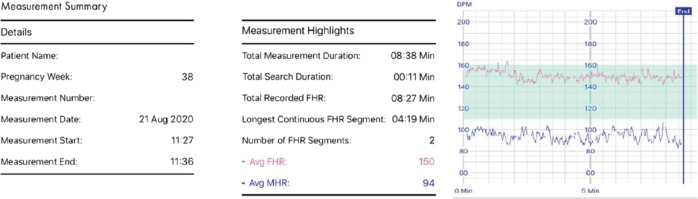
Sample data output from the fetal heart rate (FHR) monitoring system. MHR, maternal heart rate.

The system includes a smartphone-based interface that displays the FHR trace and calculated parameters (average FHR and MHR using beat-to-beat calculation, duration of FHR trace, duration of search time, and longest continuous FHR segment) on a Bluetooth-connected smartphone and then uploads it to a clinical management system connected to the clinic. Recordings can be observed simultaneously in the clinic to enable an immediate medical response. The user and the supervising clinic are immediately notified of all measurements falling outside of nominated safe ranges. In addition, a printable, storable recording of the fetal and MHRs are produced for onsite or remote review (Fig. 6).

The system complies with the HIPAA (Health Insurance Portability and Accountability Act) policies on privacy and transmission capabilities^3^.

System specifications and safety claims are presented in supplementary table 1.

### Data collection

This was a prospective, single-centre, unblinded clinical study conducted in two phases. Participants were recruited as a convenience sample from the antenatal clinic of a large metropolitan hospital in Western Australia. No aspect of the study interfered with clinical care.

Women aged 18 years or older with a singleton pregnancy of at least 26 weeks gestation were approached to participate in the study. Women who could not read English, had a skin rash or condition on the abdomen that could be irritated by ultrasound gel or had a pacemaker, or other implantable electronic devices were excluded. Women who did not have access to a smartphone or internet connectivity were unable to participate in the home-recording phase. Enrolment was undertaken by research nurses and clinic midwives who explained the study and obtained written informed consent^3^.

Phase 1 was conducted from November 2020 to July 2021 in the recruiting site’s antenatal clinic and inpatient ward. Clinicians performed CTG recordings using a Phillips Avalon FM20 or Avalon FM30 machine. After placement of the CTG in the prime abdominal location, the HBM was placed in the next best position to record the FHR simultaneously. Forty of the 63 enrolled participants used a standard disposable belt to stabilize the HBM. Twenty-three participants held the device in-situ by hand.

Phase 2 was conducted from March to August 2021 at the participants’ homes. Women performed unsupervised, self-administered fetal cardiography using the HBM. A research nurse demonstrated how to use the HBM during a 5-minute training session in the antenatal clinic. Participants were required to use the monitor unassisted at home to detect and record the FHR for longer than 20 minutes. Participants used the HBM in the self-guided mode, which uses the inbuilt position guidance system. The device was held in place using a standard disposable CTG belt. Participants were then asked to rate the heartbeat monitor for usability and learnability using the international medical standard System Usability Scale (SUS)^17–19^.

We collected data on participants’ age, gestation, height, weight, pre-pregnant body mass index (BMI, calculated as weight in kilograms divided by height in meters squared), BMI at enrolment, gravidity, parity, presence of a structural uterine abnormality, and location of the placenta, as well as data from the heartbeat monitors.

### Data Analysis

We reviewed all fetal cardiography recordings from the two phases for the following outcome measures: 1) detection of FHR (different from MHR), 2) baseline FHR, 3) longest length continuous recordings longer than 1 minute, 3) total FHR recording time, and 4) time taken to detect FHR.

**Fig. 7:**
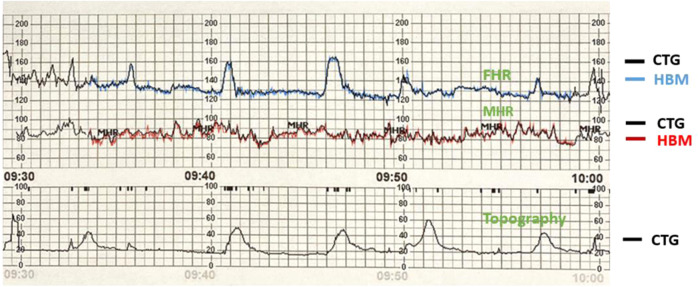
Superposition of fetal and maternal heartrate recordings collected by cardiotocography and fetal heartbeat monitor. FHR Fetal Heat rate, MHR Maternal heart rate, CTG cardiotocography.

We assessed the accuracy of the HBM against CTG by calculating paired fetal heart rate measurements. The raw data included were CTG data (4 Hz/4 per second sampling frequency) vs HBM fetal cardiography data (1 Hz/1 per second sampling frequency). Superposing CTG data and HBM data, we performed a visual comparison. The first 30 minutes (or less in cases of shorter traces) were taken for the visual superposition. An example of a superimposed HBM and CTG trace is presented in Fig. 7. Each HBM FHR data point was repeated four times for sampling frequency synchronization. To achieve optimal superposition, we calculated the Root Mean Squared Error (RMSE) between the points on each graph. Then, the graphs were incrementally shifted until optimal superposition (minimal RMSE value).

Differences in FHR (bpm) between the paired measurements were analyzed. The agreement between the HBM and stationary device was established using Bland Altman plots and 95% limits of agreement. Reliability was established using intraclass correlation coefficients using a two-way mixed-effects model. Linear regression/coefficients were calculated to detect the presence of proportional bias. The measurement comparison was deemed accurate if the 95% limits of agreement were within eight bpm. This target was selected in keeping with other accuracy studies of FHR monitors as a clinically acceptable range in which important features, such as fetal bradycardias, accelerations, and decelerations, can be recognized^8,9^.

Bland Altman analysis was performed by assessing 2 minutes of measurement pairs from each participant. The 2 minutes sample was selected as the first 2 minutes without signal loss for both the simultaneous CTG and HBM traces. One hundred and twenty data pairs (1 per second) took the average of 4 CTG measurements for each HBM measurement. Bland Altman Plots were drawn taking 2 minutes pairs of measurements of average heartbeat/bpm for each participant (23 participants without belts and 40 participants with belts).

Signal Loss Ratio (SLR) was calculated separately for the CTG and HBM fetal cardiography data. The SLR was calculated as the percentage of data points with missing FHR value in the first 30 minutes of parallel tracing (or less in cases of shorter traces). The International Federation of Gynaecology and Obstetric recommendations describe an acceptable fetal signal loss of 20%^20^.

Clinical interpretability of fetal cardiography was assessed by a senior obstetrician using the standard definitions and criteria for FHR characteristics and NST assessments (Box 1), including whether the FHR was detected (Y/N) and if the trace was of acceptable quality to determine the baseline FHR (bpm), and the presence or absence of accelerations, decelerations, and FHR variability, and the grade of FHR variability (normal/increased/ decreased) were compared.

To assess the usability and learnability of the HBM, we used the international medical standard System Usability Scale (SUS)^17^. The SUS is a 10-statement survey that evaluates the learnability, reliability, and usability of products. It has been shown to have high reliability (alpha of .91) over a wide range of interface types^18^. When evaluating the results, SUS raw scores are reported as means and 95% CIs and converted to a percentile rank (0–100) with a corresponding letter grade (A + to F), as per the SUS scoring system template (http://links.lww.com/AOG/C240, supplementary table 2).

We used the positive version of the System Usability Scale (SUS) and included an additional adjective rating scale, a single Likert scale question, that demonstrates a high correlation with overall SUS scores of 13 (http://links.lww.com/AOG/C240, supplementary table 3). Given the skewed distribution, the median and interquartile range were provided when reporting the adjectival rating scale. Participants completed the SUS questionnaires after using the HBM at home.

Subgroup analyses was conducted to evaluate the relationship between BMI, gestation, obstetric history, and placental position on outcome measures for Phase 1 and Phase 2 participants and for the subgroup of women who were beyond 28 weeks of gestation (in which cardiotocography monitoring is typically performed). The relationship with clinical features was assessed using nonparametric tests due to skewed distributions (Kruskal-Wallis and Mann-Whitney U).

### Reporting summary

Further information on research design is available in the Nature Research Reporting Summary linked to this article.

## Supplementary information

Supplementary Files

Reporting Summary

## Data Availability

The datasets generated during and/or analysed during the current study are available from the corresponding author on reasonable request.

## Code availability

All data were collated using Microsoft Excel (2016) and analyzed using SPSS Version 27.
