# Supplementary material for: Accuracy, interpretability and usability study of a wireless self-guided fetal heartbeat monitor compared to cardiotocography
Source: NPJ Digit Med. 2022 Nov 3;5:167. doi: 10.1038/s41746-022-00714-6 (PMC9630800; doi:10.1038/s41746-022-00714-6)
Supplement: Supplementary file 1 — Supplementary Files [file 41746_2022_714_MOESM1_ESM.pdf]

Supplementary Files

Supplementary table 1

HeraBEAT system specification and safety claims.

Characteristic	Measure	Specifications
Safety	Complies with	IEC/EN 60601-1, 60601-1-2, 60601-1-11, 60601-2-37
Classification	Antielectric shock type	Class II electrical device when AC/DC adapter connected. Otherwise, internally powered equipment
	Antielectric shock degree	Type BF equipment
	Degree of protection against harmful ingress of water	IP22; Protection against falling drops of water when unit is tilted 15º
Physical characteristics	Device size	88 x 37 mm; 3.5 x 1.5 inches (Diameter × Height, ± 0.08 inches
	Device weight	Approximately 4.58 ounces
Operating environment	Temperature	From 41°F up to 104°F
	Humidity	From 5% up to 90% RH (noncondensing)
Storage/transport environment	Temperature	From -4°F up to 140°F
	Humidity	From 5% up to 95% (noncondensing)
	Light intensity	No direct sunlight
FHR performance	Pregnancy week	12 to 42
	FHR measuring range; accuracy; resolution	50 to 240 bpm; ± 2 bpm; 1 bpm
	MHR measuring range; accuracy; resolution	45 to 240 BPM; ± 2% or 1 bpm, whichever is greater; 1 bpm
Auto acquisition stop	NA	5 minutes of successful measurement
Recommended ultrasound transmission gel	NA	Aquasonic 100 Ultrasound Transmission Gel (Parker Laboratories, Fairfield, NJ)
Power consumption	NA	<2 W
Rechargeable lithion-ion battery	Nominal capacity	3.7 V DC, 1250 mAH
	Continuous work time	4 hours (with a new battery)
	Power input	5 V DC, >0.3 A
	Charge time	4 hours
Ultrasound (NEMA/FDA)	Nominal frequency	2 MHz ± 10%
	Ultrasonic output power (P)	70 mW
	Peak rarefactional pressure (p_r)	0.03 MPa
	Ultrasonic output intensity (I_{sata})	≤20 mW/cm^2
	Mechanical index (MI)	0.02
	Thermal index (TIS; TIB)	0.26; 0.7
	Measurement mode	Continuous wave ultrasound doppler
	Effective radiating area of transducer	4.9 ± 0.5 cm^2

BLE specification	Frequency band of transmission	2.4–2.5 GHz<br>Channels (2 MHz spacing)<br>3 advertising channels @ 2402, 2426, 2480 Mh<br>36 data channels
	Frequency characteristics of the modulation	DSSS: GFSK (modulation index=0.5)
	Maximum RF input	-10 dBm
	Typical receive sensitivity	-94 dBm
	Maximum RF Tx output power	+4 dBm

HeraBEAT safety claims:

- HeraBEAT works at low voltage (5 V), which is supplied from an internal rechargeable battery (tested per IEC 60601-1).
- HeraBEAT device material is isolated and made of electric nonconducting material. In addition, the device does not operate while charging.
- HeraBEAT transmits ultrasonic energy at a maximum intensity of 20 mW/c “^{2}, according to IEC 60601-2-37 “Medical electrical equipment – Part 2-37: Particular requirements for the safety of ultrasonic medical diag “ostic and monitoring equipment.”
- The device turns off if not connected to the mobile app for several seconds.
- All materials are biocompatible and approved for use on the skin surface.
- HeraBEAT controls the temperature level inside the device to assure that the device temperature remains below the safe temperature limit. In addition, a built-in test (BIT) is implemented to verify the correct functioning of the temperature sensor.
- The device conforms to risk management best practices according to ISO 14971:2007 – Medical Devices – Application of Risk Management to Medical Devices.

Supplementary table 2

Conversion table for System Usability Scale raw scores into percentile and grades.

SUS Score	Percentile	Grade
84.1–100	96–100	A+
80.8–84.0	90–95	A
78.9–80.7	85–89	A-
77.2–78.8	80–84	B+
74.1–77.1	70–79	B
72.6–74.0	65–69	B-
71.1–72.5	60–64	C+
65.0–71.0	41–59	C
62.7–64.9	35–40	C-
51.7–62.6	15–34	D
<51.7	0–14	F

Supplementary table 3

All positive version of the System Usability Scale and the adjectival enhancement question used in the study.

Please mark the box that reflects your immediate response to each statement. Don't think too long about each statement. Please make sure you respond to every statement. **If you don't know how to respond, just mark box '3.'**					
	Strongly disagree				***Strongly agree***
**1.** I think I would like to use this system frequently.	1	2	3	4	5
**2.** I found this system to be simple.	1	2	3	4	5
**3.** I thought this system was easy to use.	1	2	3	4	5
**4**. I think I could use this system without the support of a technical person.	1	2	3	4	5
**5.** I found the various functions of this system were well integrated.	1	2	3	4	5
**6.** I thought there was a lot of consistency in this system.	1	2	3	4	5
**7.** I imagine most people would learn to use it very quickly.	1	2	3	4	5
**8.** I found it very intuitive.	1	2	3	4	5
**9**. I felt very confident using this system	1	2	3	4	5
**10.** I could use this system without having to learn anything new.	1	2	3	4	5

Adjectival assessment: Overall, I would rate the user-friendliness of HBM as….	Worst imaginable	Awful	Poor	Okay	Good	Excellent	Best imaginable
	1	2	3	4	5	6	7

Supplementary table 4

Factors related to clinical outcomes (Phase 1). Gestational age not included as all participants are in Trimester 3. *p* values derived using Kruskal-Wallis and Mann-Whitney U

User		Clinician Administered	
Site of recording		Antenatal Clinic (Phase 1) (n = 63)	
		Median (IQR)	*p* value
Time to 1st detection of FHR (**Seconds**)			
BMI at enrolment (kg/m^2)	<23.5	29 (14 – 44)	0.78
	23.5 to < 30	14 (14 – 25)	
	30 to <35	14 (14 – 25.6)	
	35 to <45	14 (15 – 18)	
BMI at enrolment ≤ 35 (kg/m2)	< 35	14 (14 – 15)	0.86
	≥ 35	14 (14 – 18)	
Anterior Placenta Location	Yes	14 (14 - 14)	0.09
	No	14 (14 -1 6)	
Continuous FHR Trace Duration (**Minutes**)			
BMI at enrolment (kg/m^2)	<23.5	12.7 (3.1 – 22.2)	0.44
	23.5 to < 30	8.8 (6.8 – 15.5)	
	30 to <35	7.1 (4.3 – 11.2)	
	35 to <45	10.1 (6.9 – 13.2)	
BMI at enrolment ≤ 35 (kg/m2)	< 35	7.9 (5.1 – 13.4)	0.48
	≥ 35	10.1 (6.9 – 13.2)	
Anterior Placenta Location	Yes	7.3 (4.7 – 12.1)	0.27
	No	9.2 (6.5 – 16.2)	
Signal Loss (%)			
BMI at enrolment (kg/m^2)	<23.5	10.0 (0.4 – 19.6)	0.39
	23.5 to < 30	5.8 (3.3 – 10.5)	
	30 to <35	11.3 (4.2 – 16.9)	
	35 to <45	12.4 (4.8 – 20.2)	
BMI at enrolment ≤ 35 (kg/m2)	< 35	8.2 (3.8 – 16.1)	0.28
	≥ 35	12.4 (4.8 – 30.9)	
Anterior Placenta Location	Yes	9.3 (4.5 – 13.4)	0.93
	No	6.6 (2.7 – 21.3)	

Supplementary table 5

Factors related to clinical outcomes (Phase 2). *p* values derived using Kruskal-Wallis and Mann-Whitney U

User		Clinician Administered	
Site of recording		Participant's Home (Phase 2) n = 34	
		Median (IQR)	*p* value
Time to 1st detection of FHR (Seconds)			
BMI at enrolment (kg/m^2)	23.5 to < 30	30 (15 – 60)	0.053
	30 to <35	15 (15 – 15)	
	35 to <45	15 (15 – 15)	
BMI at enrolment ≤ 35 (kg/m2)	< 35	15 (15 – 30)	0.45
	≥ 35	15 (15 – 75)	
Anterior Placenta Location	Yes	15 (15 – 30)	0.92
	No	15 (15 – 75)	
Gestation Age	2nd Trimester (Week 14-26)	382.5 (15 – 750)	0.33
	3rd Trimester (Week 27+)	15 (15 – 30)	
Continuous FHR Trace Duration			
BMI at enrolment (kg/m^2)	23.5 to < 30	8.8 (4.7 – 13.9)	0.59
	30 to <35	6.4 (3.1 – 8.74)	
	35 to <45	7.7 (5.3 – 14.2)	
BMI at enrolment ≤ 35 (kg/m2)	< 35	6.4 (4.6 – 9.8)	0.40
	≥ 35	7.7 (6.1 – 12.8)	
Anterior Placenta Location	Yes	7.6 (4.4 – 13.6)	0.76
	No	6.5 (5.0 – 9.4)	
Gestation Age	2nd Trimester (Week 14-26)	14.8 (8.6 – 20.9)	0.12
	3rd Trimester (Week 27+)	6.5 (4.6 -11.3)	
Signal Loss (%)			
BMI at enrolment (kg/m^2)	23.5 to < 30	2.6 (1.3 – 6.6)	0.17
	30 to <35	6.0 (2.7 – 9.9)	
	35 to <45	6.7 (1.5 – 8.4)	
BMI at enrolment ≤ 35 (kg/m2)	< 35	4.3 (2.1 – 9.7)	0.99
	≥ 35	6.7 (1.5 – 8.4)	
Anterior Placenta Location	Yes	2.9 (1.0 – 9.5)	0.31
	No	6.6 (3.3 – 9.7)	
Gestation Age	2nd Trimester (Week 14-26)	1.4 (1.0 – 1.7)	0.09
	3rd Trimester (Week 27+)	5.9 (2.4 – 9.6)	
