# Supplementary material for: Accuracy, interpretability and usability study of a wireless self-guided fetal heartbeat monitor compared to cardiotocography
Source: NPJ Digit Med. 2022 Nov 3;5:167. doi: 10.1038/s41746-022-00714-6 (PMC9630800; doi:10.1038/s41746-022-00714-6)
Supplement: Supplementary file 2 — Reporting Summary [file 41746_2022_714_MOESM2_ESM.pdf]

nature portfolio

Corresponding author(s): Dr Paul Porter

Last updated by author(s): 2022/09/09

Reporting Summary

Nature Portfolio wishes to improve the reproducibility of the work that we publish. This form provides structure for consistency and transparency in reporting. For further information on Nature Portfolio policies, see our Editorial Policies and the Editorial Policy Checklist.

Statistics

For all statistical analyses, confirm that the following items are present in the figure legend, table legend, main text, or Methods section.

n/a	Confirmed	
☐	☒	The exact sample size (*n*) for each experimental group/condition, given as a discrete number and unit of measurement
☐	☒	A statement on whether measurements were taken from distinct samples or whether the same sample was measured repeatedly
☐	☒	The statistical test(s) used AND whether they are one- or two-sided<br>*Only common tests should be described solely by name; describe more complex techniques in the Methods section.*
☐	☒	A description of all covariates tested
☐	☒	A description of any assumptions or corrections, such as tests of normality and adjustment for multiple comparisons
☐	☒	A full description of the statistical parameters including central tendency (e.g. means) or other basic estimates (e.g. regression coefficient) AND variation (e.g. standard deviation) or associated estimates of uncertainty (e.g. confidence intervals)
☐	☒	For null hypothesis testing, the test statistic (e.g. *F*, *t*, *r*) with confidence intervals, effect sizes, degrees of freedom and *P* value noted<br>*Give P values as exact values whenever suitable.*
☒	☐	For Bayesian analysis, information on the choice of priors and Markov chain Monte Carlo settings
☒	☐	For hierarchical and complex designs, identification of the appropriate level for tests and full reporting of outcomes
☐	☒	Estimates of effect sizes (e.g. Cohen's *d*, Pearson's *r*), indicating how they were calculated

Our web collection on statistics for biologists contains articles on many of the points above.

Software and code

Policy information about availability of computer code

Data collection	N/A
Data analysis	All data were analyzed using SPSS Version 27.

For manuscripts utilizing custom algorithms or software that are central to the research but not yet described in published literature, software must be made available to editors and reviewers. We strongly encourage code deposition in a community repository (e.g. GitHub). See the Nature Portfolio guidelines for submitting code & software for further information.

Data

Policy information about availability of data

All manuscripts must include a data availability statement. This statement should provide the following information, where applicable:

- Accession codes, unique identifiers, or web links for publicly available datasets
- A description of any restrictions on data availability
- For clinical datasets or third party data, please ensure that the statement adheres to our policy

The datasets generated during and/or analysed during the current study are available from the corresponding author on reasonable request.

Human research participants

Policy information about studies involving human research participants and Sex and Gender in Research.

Reporting on sex and gender	This study included only pregnant participants and the term "sex" was used as a consequence.
Population characteristics	Presented in tabular form in the manuscriopt
Recruitment	Convenience sampling
Ethics oversight	Ramsay Health Care HREC, NSW and Victoria

Note that full information on the approval of the study protocol must also be provided in the manuscript.

Field-specific reporting

Please select the one below that is the best fit for your research. If you are not sure, read the appropriate sections before making your selection.

☒ Life sciences ☐ Behavioural & social sciences ☐ Ecological, evolutionary & environmental sciences

For a reference copy of the document with all sections, see nature.com/documents/nr-reporting-summary-flat.pdf

Life sciences study design

All studies must disclose on these points even when the disclosure is negative.

Sample size	97 participants were recruited as a convenience sample from the antenatal clinic of a large metropolitan hospital in Western Australia. 63 participants were in Phase 1 during antenatal clinical appointment and 34 were at home.
Data exclusions	Women aged 18 years or older with a self-reported singleton pregnancy of at least 12 weeks of gestation were approached to participate in the study. Women who could not read English, had a skin rash or condition on the abdomen that could be irritated by ultrasound gel, or had a pacemaker or other implantable electronic devices were excluded. Women who did not have access to a smartphone or Internet connectivity were unable to participate in the home-recording phase.
Replication	Clinical interpretability of fetal cardiography was assessed by a senior obstetrician using the standard definitions and criteria for FHR characterisitcs and non-stress test assessments. A research nurse explained and demonstrated how to use the HBM during a 5-minute training session in the antenatal clinic.
Randomization	Not applicable.
Blinding	Not applicable.

Behavioural & social sciences study design

All studies must disclose on these points even when the disclosure is negative.

Study description	*Briefly describe the study type including whether data are quantitative, qualitative, or mixed-methods (e.g. qualitative cross-sectional, quantitative experimental, mixed-methods case study).*
Research sample	*State the research sample (e.g. Harvard university undergraduates, villagers in rural India) and provide relevant demographic information (e.g. age, sex) and indicate whether the sample is representative. Provide a rationale for the study sample chosen. For studies involving existing datasets, please describe the dataset and source.*
Sampling strategy	*Describe the sampling procedure (e.g. random, snowball, stratified, convenience). Describe the statistical methods that were used to predetermine sample size OR if no sample-size calculation was performed, describe how sample sizes were chosen and provide a rationale for why these sample sizes are sufficient. For qualitative data, please indicate whether data saturation was considered, and what criteria were used to decide that no further sampling was needed.*
Data collection	*Provide details about the data collection procedure, including the instruments or devices used to record the data (e.g. pen and paper, computer, eye tracker, video or audio equipment) whether anyone was present besides the participant(s) and the researcher, and whether the researcher was blind to experimental condition and/or the study hypothesis during data collection.*
Timing	*Indicate the start and stop dates of data collection. If there is a gap between collection periods, state the dates for each sample cohort.*

Data exclusions	*If no data were excluded from the analyses, state so OR if data were excluded, provide the exact number of exclusions and the rationale behind them, indicating whether exclusion criteria were pre-established.*
Non-participation	*State how many participants dropped out/declined participation and the reason(s) given OR provide response rate OR state that no participants dropped out/declined participation.*
Randomization	*If participants were not allocated into experimental groups, state so OR describe how participants were allocated to groups, and if allocation was not random, describe how covariates were controlled.*

Ecological, evolutionary & environmental sciences study design

All studies must disclose on these points even when the disclosure is negative.

Study description	*Briefly describe the study. For quantitative data include treatment factors and interactions, design structure (e.g. factorial, nested, hierarchical), nature and number of experimental units and replicates.*
Research sample	*Describe the research sample (e.g. a group of tagged Passer domesticus, all Stenocereus thurberi within Organ Pipe Cactus National Monument), and provide a rationale for the sample choice. When relevant, describe the organism taxa, source, sex, age range and any manipulations. State what population the sample is meant to represent when applicable. For studies involving existing datasets, describe the data and its source.*
Sampling strategy	*Note the sampling procedure. Describe the statistical methods that were used to predetermine sample size OR if no sample-size calculation was performed, describe how sample sizes were chosen and provide a rationale for why these sample sizes are sufficient.*
Data collection	*Describe the data collection procedure, including who recorded the data and how.*
Timing and spatial scale	*Indicate the start and stop dates of data collection, noting the frequency and periodicity of sampling and providing a rationale for these choices. If there is a gap between collection periods, state the dates for each sample cohort. Specify the spatial scale from which the data are taken*
Data exclusions	*If no data were excluded from the analyses, state so OR if data were excluded, describe the exclusions and the rationale behind them, indicating whether exclusion criteria were pre-established.*
Reproducibility	*Describe the measures taken to verify the reproducibility of experimental findings. For each experiment, note whether any attempts to repeat the experiment failed OR state that all attempts to repeat the experiment were successful.*
Randomization	*Describe how samples/organisms/participants were allocated into groups. If allocation was not random, describe how covariates were controlled. If this is not relevant to your study, explain why.*
Blinding	*Describe the extent of blinding used during data acquisition and analysis. If blinding was not possible, describe why OR explain why blinding was not relevant to your study.*

Did the study involve field work? ☐ Yes ☒ No

Reporting for specific materials, systems and methods

We require information from authors about some types of materials, experimental systems and methods used in many studies. Here, indicate whether each material, system or method listed is relevant to your study. If you are not sure if a list item applies to your research, read the appropriate section before selecting a response.

Materials & experimental systems

n/a	Involved in the study	
☒	☐	Antibodies
☒	☐	Eukaryotic cell lines
☒	☐	Palaeontology and archaeology
☒	☐	Animals and other organisms
☐	☒	Clinical data
☒	☐	Dual use research of concern

Methods

n/a	Involved in the study	
☒	☐	ChIP-seq
☒	☐	Flow cytometry
☒	☐	MRI-based neuroimaging

Clinical data

Policy information about clinical studies

All manuscripts should comply with the ICMJE guidelines for publication of clinical research and a completed CONSORT checklist must be included with all submissions.

Clinical trial registration	Clinical trial registration: Australian New Zealand Clinical Trial Registry (ANZCTR) - Trial Id: ACTRN12620000739910.
Study protocol	*Full protocol is available on request from the authors*
Data collection	This was a prospective, single-center, unblinded clinical study conducted in two phases. Participants were recruited as a convenience sample from the antenatal clinic of a large metropolitan hospital in Western Australia.<br><br>Phase 1 was conducted from November 2020 to July 2021 in the clinic and inpatient ward of the recruiting site. Clinicians performed CTG recordings using a Phillips Avalon FM20 or Avalon FM30 machine. After placement of the CTG in the prime abdominal location the HBM was placed in the next best position by a research nurse to record the FHR simultaneously. 40 of the 63 enrolled participants used a standard disposable belt to stabilize the HBM. In addition, 23 participants held the device in-situ by hand.<br><br>Phase 2 was conducted from March to August 2021, at the participants' home, where women performed unsupervised, self-administered fetal cardiography using the HBM. A research nurse explained and demonstrated how to use the HBM during a 5-minute training session in the antenatal clinic. Participants were required to use the monitor unassisted at home to detect and record the FHR for longer than 20 minute. Participants used the HBM in the self-guided mode, which uses the inbuilt position guidance system. The device is activated and placed below the umbilicus, as directed by the smartphone interface, to a position dependent on pregnancy gestation. The device continues to self-direct positioning using audio instructions until two distinct heart rates (FHR and MHR) are detected. The device was held in place using a standard disposable CTG adaptor. Participants were then asked to rate the heartbeat monitor for usability and learnability using the international medical standard System Usability Scale (SUS). de Mooij et al., 2015.
Outcomes	Primary outcome: Comparison of accuracy and the clinical interpretability 20-minute fetal cardiography examination obtained using a wireless mobile self-guided foetal heartbeat monitor to one obtained from a traditional CTG stationary device when used in the clinic environment.<br>We reviewed all fetal cardiography recordings from the two phases for the following outcome measures: 1) detection of FHR (different from maternal heart rate), 2) baseline FHR, 3) longest length continuous recordings longer than 1 minute, 3) total FHR recording time, and 4) time taken to detect FHR.<br>We assessed the accuracy of the HBM against CTG by calculating paired foetal heart rate (FHR) measurements. The raw data included were CTG data (4HZ/4 per second sampling frequency) vs HBM fetal cardiography data (1HZ/1 per second sampling frequency).<br><br>Differences in FHR (bpm) between the paired measurements were analyzed. The agreement between the HBM and stationary device was established using Bland Altman plots and 95% limits of agreement. Reliability was established using intraclass correlation coefficients using a two-way mixed-effects model. Linear regression/coefficients were calculated to detect presence of proportional bias.<br><br>Signal Loss Ratio (SLR) was calculated separately for the CTG and HBM fetal cardiography data. The SLR was calculated as the percentage of data points with missing FHR value in the first 30 minutes of parallel tracing (or less in cases of shorter traces). The International Federation of Gynaecology and Obstetric recommendations describe an acceptable fetal signal loss of 20%.<br><br>Clinical interpretability of fetal cardiography was assessed by a senior obstetrician using the standard definitions and criteria for FHR characterisitcs and NST assessments, including whether the FHR was detected, (Y/N) and if the trace was of an acceptable quality to determine the baseline FHR (bpm), and the presence or absence of accelerations, decelerations, and FHR variabil<br><br>Secondary outcomes include evaluation of HBM in terms of clinical utility, usability and learnability when used by women, unsupervised, at home by applying the international medical standard System Usability Scale (SUS). The SUS is a 10-statement survey that evaluates the learnability, reliability, and usability of products. It has been shown to have high reliability (alpha of .91) over a wide range of interface types.Lewis et al., 2018 When evaluating the results, SUS raw scores are reported as means and 95% CIs, and converted to a percentile rank (0–100) with a corresponding letter grade (A+ to F), as per the SUS scoring system templatez.
